# 183. Combination Antibiotic Therapy vs Monotherapy for Methicillin Resistant *Staphylococcus aureus* Endocarditis

**DOI:** 10.1093/ofid/ofad500.256

**Published:** 2023-11-27

**Authors:** James M Crosby, Bobbi Jo Stoner, Kelli Cremeans, Sami El-Dalati

**Affiliations:** University of Kentucky, Lexington, Kentucky; University of Kentucky, Lexington, Kentucky; University of Kentucky, Lexington, Kentucky; University of Kentucky, Lexington, Kentucky

## Abstract

**Background:**

Mortality for methicillin-resistant Staphylococcus aureus (MRSA) bacteremia and endocarditis remains unacceptably high. It is unclear whether combination antibiotic therapy results in improved clinical outcomes, which antimicrobials are most efficacious and when to initiate a combination regimen. We retrospectively evaluated patients over 1-year with MRSA endocarditis who were treated with either monotherapy with vancomycin or daptomycin or combination therapy with daptomycin and ceftaroline (D/C) or vancomycin and ceftaroline (V/C).

**Methods:**

Patient cases were identified from the University of Kentucky’s endocarditis team (Figure 1). Demographic variables and clinical outcomes data were collected and reviewed by 2 investigators. Data analysis was conducted using Chi-Square tests and one-way ANOVA assessments. The primary outcome was in-hospital mortality. Secondary outcomes included: time to blood culture clearance and 90-day mortality.

**Figure:**
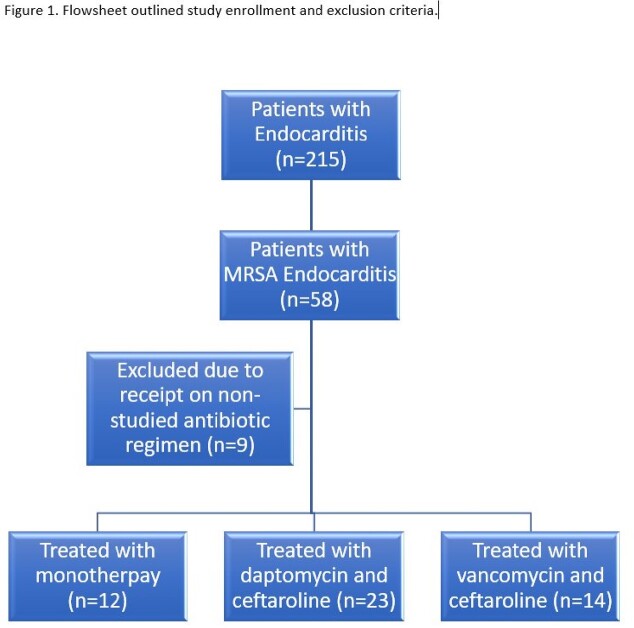

**Results:**

Between September 7th, 2021 and September 6th, 2022 we identified 49 patients with MRSA endocarditis treated with the designated regimens. There were no significant demographic differences between the study groups (Table 1). Twelve patients were treated with monotherapy, 23 patients were treated with D/C and 14 patients were treated with V/C. In-hospital mortality was 8.3% in the monotherapy group, 11.6% in the V/C group and 17.4% in the D/C group (p=0.60; Table 2). Average time to initiating combination therapy was after 4.7 days of bacteremia in the V/C group and 5.2 days in the D/C group (p=0.55). Average duration of bacteremia was 4.3 days in the monotherapy arm. Patients who received D/C cleared blood cultures after 3 days of combination therapy compared to 4.3 days in the V/C group (p=0.26). Receipt of D/C within 72 hours of the first positive blood cultures was associated with the lowest in-hospital and 90-day mortality across all groups, but this was not statistically significant (Table 3).

**Figure:**
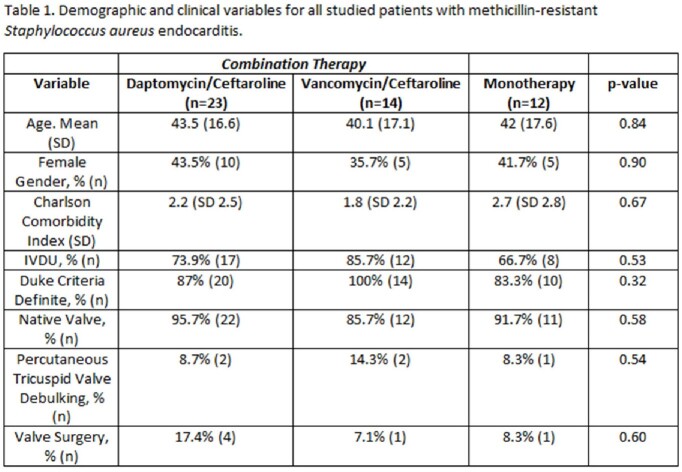

**Figure:**
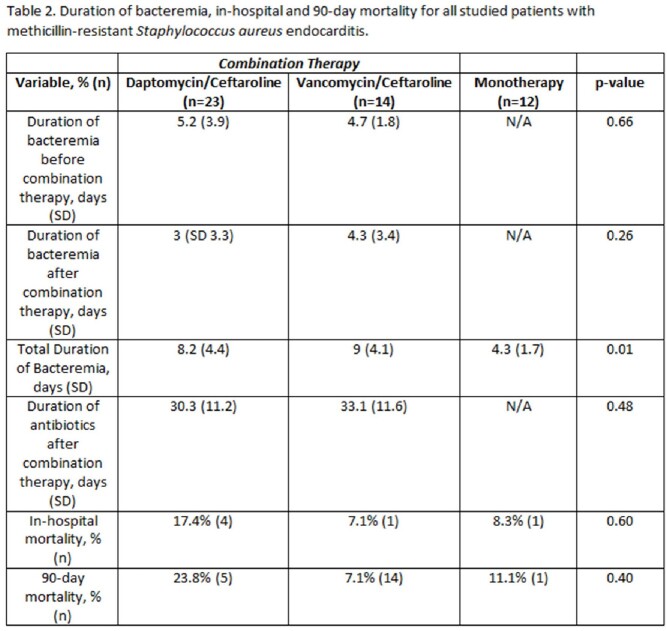

**Figure:**
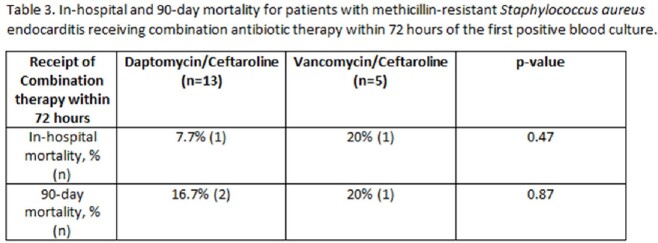

**Conclusion:**

There was no significant difference in in-hospital or 90-day mortality between patients treated with monotherapy, D/C or V/C. Further studies of larger populations are required to identify which treatment approach results in the best clinical outcomes for patients with MRSA endocarditis.

**Disclosures:**

**All Authors**: No reported disclosures

